# Complete genome assembly of *Yersinia alsatica* SCPM-O-B-7604

**DOI:** 10.1128/mra.01102-24

**Published:** 2025-04-15

**Authors:** Angelina A. Kislichkina, Angelika A. Sizova, Yury P. Skryabin, Viktor I. Solomentsev, Mikhail E. Platonov, Svetlana V. Dentovskaya, Andrey P. Anisimov

**Affiliations:** 1 State Research Center for Applied Microbiology and Biotechnology (SRCAMB), Obolensk, Russia; 2 Pushchino State Institute of Natural Science 555407, Pushchino, Russia; University of Southern California, Los Angeles, California, USA

**Keywords:** *Yersinia alsatica*, complete genome

## Abstract

We report the first published complete genome assembly of *Yersinia alsatica* SCPM-O-B-7604, the strain belonging to the new species of genus *Yersinia* described by Le Guern et al. in 2020.

## ANNOUNCEMENT

The genus *Yersinia*, a member of the family *Enterobacteriaceae*, is currently composed of 26 species (1). Three of them, *Yersinia pestis*, *Yersinia pseudotuberculosis*, and *Yersinia enterocolitica*, are well-known human pathogens (2). The remaining species are considered non-pathogenic to humans and have been investigated significantly less (3). Now the taxonomy of the genus *Yersinia* is evolving dynamically, and several novel species were recognized during research works (4, 5). Species of *Yersinia alsatica* were described by Le Guern et al. in 2020 (6).

In this report, we announce the complete genome assembly of *Y. alsatica* SCPM-O-B-7604 (isolation source is unknown). The strain has been stored in the State Collection of Pathogenic Microorganisms and Cell Cultures of SRCAMB and was originally identified as *Yersinia frederiksenii*, confirmed by biochemical identification tests (ENTEROtest 24, Erba LaChema s.r.o., Brno, CZ) and MALDI Biotyper identification (Bruker Daltonik GmbH, Germany) (7). The stocks of strains were stored at –70°C in a cryoprotective medium (20% glycerol). Bacteria were grown at 37°C on 20 mL of solid Nutrient Medium No.1 (Obolensk, Russia) for 24 hours. A few colonies were picked for DNA extraction using the DNA minikit (BioFact, Daejeon, Republic of Korea) following the manufacturer’s instructions. The extracted DNA was used for both Illumina libraries and Nanopore. Any size selection of DNA was not performed during the Nanopore sequencing.

Genome sequencing was carried out using the MinION (Oxford Nanopore, GB) and MGISeq-2000 platforms (MGI Tech Co., Ltd, China) following the manufacturer’s protocols. For MGISeq-2000, the MGIEasy FS DNA Library Prep Kit and 2000RS High-throughput Sequencing Kit PE200 were used (MGI Tech Co., Ltd, China). Rapid Barcoding Kit RBK004 and MinION Flow Cell R9.4.1 (Oxford Nanopore, GB) were used for MinION sequencing. It was run with MinKNOW software v. 18.05.5 (48 hours, 180 mV), and basecalling was performed using Guppy v. 5.0.16 (8). Default parameters were used for all software unless otherwise specified.

Up to 5,663,360 paired-end reads (8,437,778,605 bases) were generated by MGISeq-2000, and 134,780 long single reads with an average length of 1,444 bp (total count 194,628,699 bp) were generated by MinION with a total genome coverage depth of 225-fold. The hybrid genome was assembled from all reads using the software Unicycler v. 0.4.7, which included primary filtering and quality control (9). The final genome assembly has one circular chromosome, and the molecule was determined to be completed and rotated to *dnaA*. The genome size was 4,901,396 bp with a 47.5% G + C content. The chromosome was annotated with the NCBI Prokaryotic Genome Annotation Pipeline v. 6.2 (10). This strain has a total of 4,435 genes, which are composed of 4,273 protein-coding sequences, 22 rRNA genes, 83 tRNA genes, and 44 pseudogenes.

This study will help to better characterize *Y. alsatica* at the genomic level. Also, it will facilitate the understanding of genomic variability of *Yersinia* and will provide an opportunity for its comparison with other strains.

## Data Availability

The genome sequence for Y. alsatica SCPM-O-B-7604 has been deposited in GenBank under accession no. CP104006. The raw sequence reads have been deposited in the SRA under accession no. SRR21289449 (MGISeq-2000 reads) and SRR21289448 (MinION reads).
